# Supplementary material for: Causal interplay between lactose intolerance and gut microbiota: a combined bidirectional Mendelian randomization and in vivo validation study
Source: Front Nutr. 2026 Jun 1;13:1803337. doi: 10.3389/fnut.2026.1803337 (PMC13265576; doi:10.3389/fnut.2026.1803337)
Supplement: Supplementary file 1 [file Data_Sheet_1.zip › supplementary materials/Forward/leave one out cross validation/ebi-a-GCST90027469.finngen_R12_E4_LACTONAS.pdf]

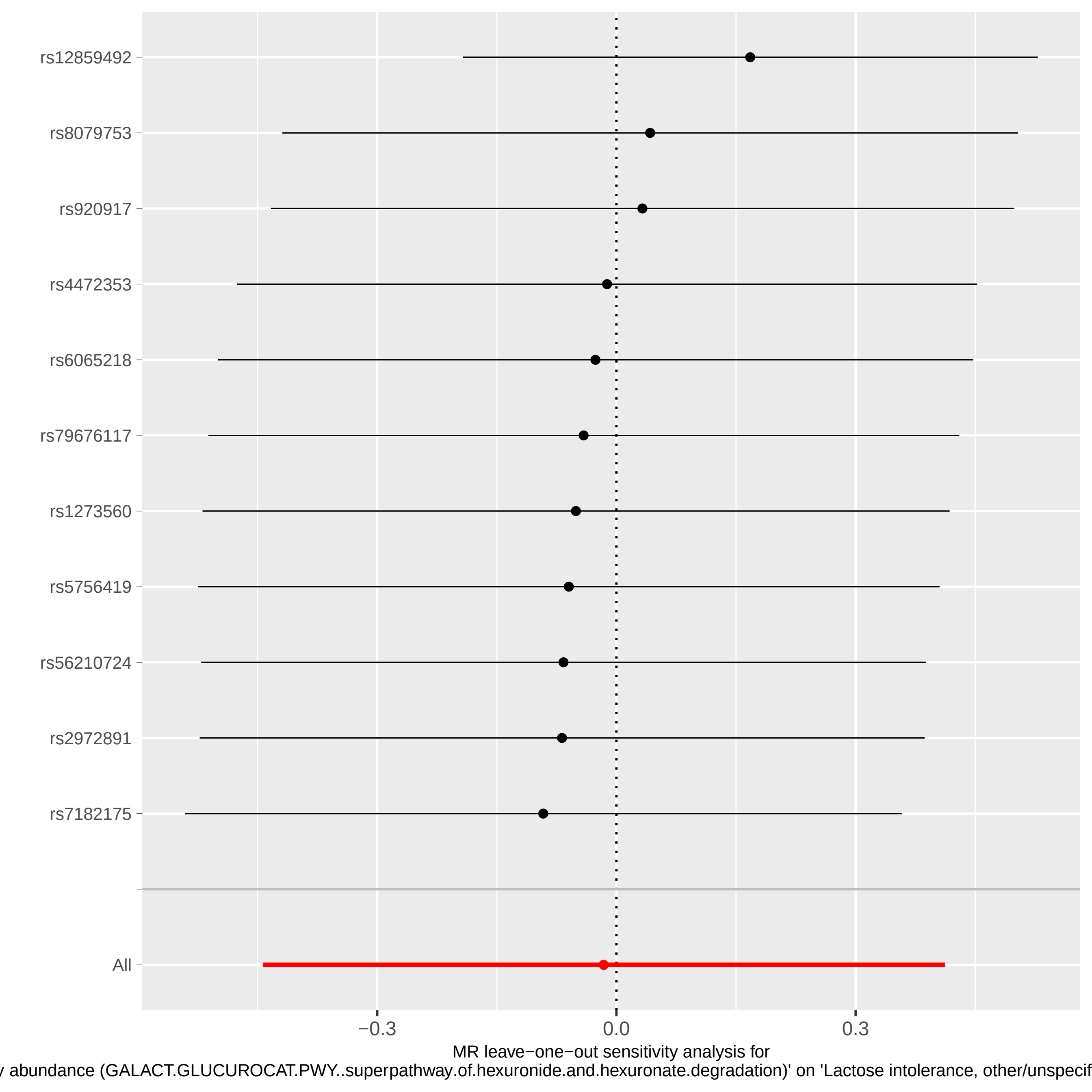
rs12859492
rs8079753
rs920917
rs4472353
rs6065218
rs79676117
rs1273560
rs5756419
rs56210724
rs2972891
rs7182175
All
−0.3
0.0
0.3
MR leave−one−out sensitivity analysis for
y abundance (GALACT.GLUCUROCAT.PWY..superpathway.of.hexuronide.and.hexuronate.degradation)' on 'Lactose intolerance, other/unspecif
